# Redox alterations of platelets and erythrocytes represent progression marker and pathogenetic determinants in Kawasaki disease

**DOI:** 10.1186/1824-7288-41-S2-A69

**Published:** 2015-09-30

**Authors:** Elisabetta Straface, Donatella Pietraforte, Lucrezia Gambardella, Domenico Del Principe, Alessandra Marchesi, Marina Viora, Isabella Tarissi De Jacobis, Alberto Villani, Walter Malorni

**Affiliations:** 1 Department of Therapeutic Research and Medicine Evaluation, Istituto Superiore di Sanità, Rome, 00161, Italy; 2 Department of Cell Biology and Neurosciences, Istituto Superiore di Sanità, Rome, 00161, Italy; 3 General Pediatric and Infectious Disease Unit, Internal Care Department, Bambino Gesù Children's Hospital, Rome, 00165, Italy

## Background

Kawasaki disease (KD) is a rare generalized systemic vasculitis of unknown etiology in which the main complication is the development of coronary artery abnormalities. Considering that an inflammation-associated systemic pro-oxidant status could play a critical pathogenetic role in KD progression [1], we evaluated some peripheral blood redox-associated parameters, including redox and aging features associated with red blood cells (RBCs) and platelets (PLT) integrity as possible pathogenetic determinants or progression markers in KD disease.

## Materials and methods

The 18 KD patients, aged between 6 and 24 mounts, were recruited from the Bambino Gesù Hospital (BGH) of Rome (Italy) and studied, after obtaining the parent informed consent, before to start therapy with intravenous immunoglobulin and aspirin. Ten age-matched healthy donors (HD) were enrolled as controls. The study was approved by the BGH Institutional Review Board.

Morphological, biophysical, biochemical and flow cytometrical methods were used to evaluate: i) reactive oxidizing species (ROS) formation and oxidative stress-related biomarkers [3-nitrotyrosine, the endothelial nitric oxide synthase inhibitor, asymmetric dymethylarginine (ADMA), the pro-oxidant enzyme myeloperoxidase (MPO)]; ii) PLT integrity and function, including PLT activation and procoagulant state (annexin V positivity); iii) RBCs homeostasis, including RBC aging markers (glycophorin A and CD47 expression, annexin V positivity).

## Results

With respect to HD, peripheral blood of KD patients showed increased levels of O_2_·, ·NO, 3-nitrotyrosine and MPO, and decreased ADMA concentration (Figure 1). In RBCs, alterations of biomarkers correlated with cell aging and death (i.e., decreased glycophorin A and CD47 expression, increased percentage of annexin V positive cells) [2] occurred. Interestingly, we found that two different PLT sub-populations seem to coexist in the KD peripheral blood (Figure 1): i) annexin V positive PLT, showing loss of mitochondrial membrane potential (considered as pro-coagulant) and ii) annexin V negative PLT, showing mitochondrial membrane hyperpolarization (non-pro-coagulant *per se*). These two sub-populations may considerably affect the coagulation cascade and inflammatory responses in KD patients [3].

## Conclusions

These results lead us to hypothesize that the oxidative/nitrative stress occurring in KD inflamed blood vessels could alter both RBCs and PLT homeostasis, resulting in a sort of premature aging in these circulating cells that could lead to anemia and the formation of blood clots. These alterations could play a pathogenetic role in the cardiovascular complications often associated with KD but, in addition, the possible use of these data as real time biomarkers of progression cannot be ruled out.
